# Effect of Erythropoietin in patients with acute myocardial infarction: five-year results of the REVIVAL-3 trial

**DOI:** 10.1186/s12872-016-0464-3

**Published:** 2017-01-21

**Authors:** Birgit Steppich, Philip Groha, Tareq Ibrahim, Heribert Schunkert, Karl-Ludwig Laugwitz, Martin Hadamitzky, Adnan Kastrati, Ilka Ott, for the Regeneration of Vital Myocardium in ST-Segment Elevation Myocardial Infarction by Erythropoietin (REVIVAL-3) Study Investigators

**Affiliations:** 1 Deutsches Herzzentrum der Technischen Universität München, Lazarettstr. 36, 80636 Munich, Germany; 2 Medizinische Klinik Klinikum rechts der Isar der Technischen Universität München, Ismaningerstr. 22, 81675 Munich, Germany

**Keywords:** Erythropoietin, Acute myocardial infarction, REVIVAL-3 trial

## Abstract

**Background:**

Erythropoietin (EPO) has been suggested to promote cardiac repair after MI. However, the randomized, double-blind, placebo controlled REVIVAL-3 trial showed that short term high dose EPO in timely reperfused myocardium does not improve left ventricular ejection fraction after 6 months. Moreover, the study raised safety concerns due to a trend towards a higher incidence of adverse clinical events as well as a increase in neointima formation after treatment with EPO. The present study therefore aimed to assess the 5-year clinical outcomes.

**Methods:**

After successful reperfusion 138 patients with STEMI were randomly assigned to receive epoetin beta (3.33×10^4^ U, *n* = 68) or placebo (*n* = 70) immediately, 24 and 48 h after percutaneous coronary intervention. The primary outcome of the present study- the combined incidence of MACE 5 years after randomization - occurred in 25% of the patients assigned to epoetin beta and 17% of the patients assigned to placebo (RR 1.5; 95% CI 0.8-3.5; *p* = 0.26). Target lesion revascularization was required in 15 patients (22.1%) treated with epoetin-ß and 9 patients (12.9%) treated with placebo (*p* = 0.15). Analysis of patients in the upper and lower quartile of baseline hemoglobin as an indirect estimate of endogenous erythropoietin levels revealed no significant impact of endogenous erythropoietin on efficiency of exogen administered epoetin-ß in terms of death and MACE.

**Conclusion:**

These long-term follow-up data show that epoetin beta does not improve clinical outcomes of patients with acute myocardial infarction.

**Trial registration:**

URL www.clinicaltrials.gov; Unique identifier NCT00390832; trial registration date October 19th 2006

## Background

Despite continually improved treatment regimens the rate of death and heart failure is still substantially high after ST-elevation myocardial infarction (STEMI) [1–3].

The extent of myocardial necrosis is a main predictor of mortality and morbidity after STEMI. Cardiac necrosis is not only determined by the myocardial ischemia itself, but also driven by secondary damage upon reperfusion, the ischemia-reperfusion-injury. While the ischemia-induced necrosis can effectively be treated by timely myocardial reperfusion using percutaneous coronary intervention (PCI), reperfusion-induced necrosis is still barely preventable [4].

Erythropoietin (Epo), a hypoxia induced hormone, has been shown to play a cardioprotective role in various experimental models of myocardial ischemia and ischemia-reperfusion via pleiotropic actions [5]. Besides stimulation of haematopoesis, Epo induces mobilization of endothelial progenitor cells and promotes neovascularization and angiogenesis [6, 7]. It also exhibits anti-apoptotic, anti-inflammatory and anti-oxidative properties in the heart [5], where cardiomyocytes and endothelial cells express functional Epo receptors [8, 9].

However, despite promising results of experimental and preclinical studies, we -like most other clinical trials- showed in the randomized, double-blind, placebo controlled REVIVAL-3 trial, that short-term, high dose epoetin beta in addition to successful PCI in STEMI does neither reduce infarct size nor improve left ventricular function at 6 months [10–12]. On the contrary we observed a trend towards a higher incidence of adverse clinical events 6 month after epoetin beta treatment as well as a significant increase in neointima formation in the erythropoietin group [13]. This raises safety concerns about the use of erythropoietin in patients with acute MI. By promoting neointima formation and imparing arterial healing, erythropoietin might affect clinical outcomes of STEMI patients over the longer term. Moreover legacy or memory effects can influence clinical prognosis even long after cessation of drug administration [14]. However clinical outcome data more than 12 month after erythropoietin therapy have never been reported in patients treated for myocardial infarction. Thus, the aim of the present trial was to assess the impact of high-dose, short term erythropoietin on long-term clinical outcomes in STEMI patients. For this purpose we extended the follow up of the REVIVAL-3 trial, which compared 3 daily IV doses of 33,000 I.U. of rhEpoetin beta administered immediately, 24 and 48 h after PCI in STEMI to placebo treatment, up to 5 years.

## Methods

### Patients and protocol

The detailed study design and main results from the REVIVAL-3 trial have been published previously [10]. In brief, the REVIVAL-3 study was a prospective, randomized, double-blind, placebo-controlled trial allocating patients with acute STEMI in a 1:1 ratio after successful primary PCI to medical treatment with either epoetin beta or placebo as a supplement to treatment according to guidelines.

To be included patients had to present with a first STEMI within 24 h of symptom onset and had to have an angiographic left ventricular ejection fraction (LVEF) of less than 50% by visual estimation in the angiogramm. The study drug was given immediately after successful PCI in the catheterization laboratory as well as 24 and 48 h after randomization. Each time, patients received either 3.33 × 10^4^ IU of recombinant human epoetin-β (NeoRecormon; F. Hoffmann-La Roche, Basel, Switzerland) or a matching placebo intravenously for 30 min. The periprocedural antithrombotic therapy consisted of 600 mg of clopidogrel orally, 500 mg aspirin, and unfractionated heparin with or without abciximab intravenously. Heparin was given as a bolus of 140 IU or 70 IU in case of additional abciximab (0.25 mg/kg body weight bolus, followed by an infusion of 0.125 μg/kg per min for 12 h). Postinterventional all patients recieved clopidogrel 75 mg twice a day for 3 days followed by 75 mg/d for at least 6 months. Aspirin 100 mg twice a day was recommended indefinitely.

The study protocol was approved by the institutional ethics committee and all patients gave written informed consent for participation in the study. The study has been registered in clinicaltrials.gov (NCT00390832).

One hundred thirty-eight patients were randomized, from January 2007 to November 2008 at the Deutsches Herzzentrum and 1st Medizinische Klinik rechts der Isar, to epoetin-ß (*n* = 68) or placebo (*n* = 70) and finally included in the present extended follow up study.

### Clinical follow-up

The pre-specified primary end point of the main REVIVAL-3 trial was LVEF 6 months after random assignment measured by MRI. Other end points included infarct size at 5 days and 6 months and clinical adverse events (death, recurrent myocardial infarction, stroke, and infarct-related artery revascularization) at 30 days and 6 months.

Epoetin did not improve LVEF or reduce infarct size at 6 months follow up. On the contrary, there was a trend toward a higher adverse event rate with erythropoietin at 6 months.

The primary outcome of interest for the current analysis was the combined incidence of major adverse cardiac events (MACE), including death, recurrent MI, stroke, coronary bypass surgery (ACVB) and target vessel revascularistion, 5 years after randomization. The incidence of the individual components of the primary end point was also assessed. Information on vital status, recurrent MI, target vessel revascularization and stroke was collected by annual telephone interviews and from hospital records. In case the patients reported cardiac symptoms during the interview, complete clinical, electrocardiogram, and laboratory examination was performed in the outpatient clinic or by the referring physician. Reinfarction was defined as the onset of recurrent symptoms of ischemia combined with new ST-segment elevations and/or a second increase of serum CK or CK-MB to at least twice the upper limit of the normal range. Target vessel revascularization was defined as PCI or bypass grafting of the infarct-related coronary artery after primary PCI.

### Statistical analysis

All data were analyzed on the basis of the intention-to-treat principle using data from all patients as randomized. Categorical data are presented as counts or proportions (%). Continuous data are presented as mean ± standard deviation. Differences between the groups were assessed using *χ* ^2^ or Fisher exact test for categorical data and *t* test for continuous data. The cumulative incidence of the composite end point during the 5-year–follow up was evaluated with the Kaplan Meier method. Survival free of adverse events was defined as the interval from randomization until the event of interest. Data for patients who did not have an event of interest were censored at the date of the last follow-up. The difference in the composite event rate between the 2 study groups was checked for significance by means of a Cox proportional hazards model, which also allowed the calculation of the respective hazard ratio with its 95% confidence interval. A 2-tailed probability value < 0.05 was considered to indicate statistical significance. All analyses were performed using S-plus statistical package (S-PLUS, In- sightful Corp., Seattle, Washington).

## Results

All 138 patients enrolled in the REVIVAL-3 trial were included in the present extend follow up study. All had received the randomly assigned medication: 68 epoetin-ß and 70 placebo. One hundred thirty-four patients (97%) completed the 5-years follow up, while 4 patients were lost to follow up. Detailed baseline characteristics of the patients have been published previously and were similarly distributed in the two treatment groups. Table 1 summarizes some key data of the study population.

**Table 1:** Key characteristics of the study population

	Epoetin-ß (*n* = 68)	Placebo (*n* = 70)
Age, mean y (±SD)	59.1 (13.0)	62.1 (12.3)
Women, n(%)	12 (18)	18 (26)
Body mass index, mean (±SD)	28 (4)	27 (4)
Diabetes, n(%)	11 (16)	10 (14)
Current smoker, n(%)	29 (43)	30 (43)
Multivessel disease, n(%)	42 (62)	50 (71)
Angiographic LVEF, mean % (±SD)	46 (8)	46 (8)
Infarct related coronary artery, n(%)		
LAD	34 (50)	31 (44)
RCA	19 (33.9)	18 (31.0)
LCX	7 (12.5)	12 (20.7)
LMCA	0	1 (1)
Initial TIMI flow grade, n(%)		
0	35 (52)	41 (59)
1	11 (16)	11 (14)
2	20 (29)	15 (21)
3	2 (3)	4 (6)
Final TIMI flow grade, n(%)		
1	0	1 (1)
2	5 (7)	6 (9)
3	63 (93)	63 (90)
Type of intervention, n(%)		
Bare metal stent	3 (4)	3 (4)
Drug-eluting stent	63 (93)	66 (95)
Balloon angioplasty	2 (3)	1 (1)
Creatine kinase-MB max, U/L (range)	201 (121–450)	213 (124–312)
Symtom onset to PCI, min (range)	252 (175–413)	253 (165–457)
Hemoglobin max, mean g/dl (±SD)	14.8 (1.6)	15 (1.3)

The mean age of the patients was 59.1 (±13.0) years in the epoetin-ß group and 62.1 (±12.3) years in the control group, with a proportion of males of 82% versus 74%. The median time from symptom onset to PCI was 252 (interquartile range 175–413) minutes in patients receiving epoetin-ß and 253 (interquartile range 165–457) minutes in patients in the control group. Baseline angiographic LVEF was 46% in both groups, indicating substantial myocardial infarction. The majority of patients presented with multi-vessel-disease (62% versus 71%) and was treated with drug-eluting stents (93% versus 95%). Although epoetin-ß induced an increase in circulating reticulocytes 5 days after random assignment (11.3 ± 3.8×10^4^/μl versus 10.9 ± 4.18×10^4^/μl; *p* = 0.563 to 34.2 ± 9.58×10^4^/μl versus 16.8 ± 6.58×10^4^/μl; *p* = 0.001) and a rise in the maximal platelet count (265 ± 70×10^9^/l versus 232 ± 74×10^9^/l, *P* = 0.011), it was not associated with a rise in maximal hemoglobin levels (14.8 ± 1.6 mg/dl versus 15 ± 1.3 mg/dl, *P* = 0.593).

### Clinical outcome

**Fig. 1:**
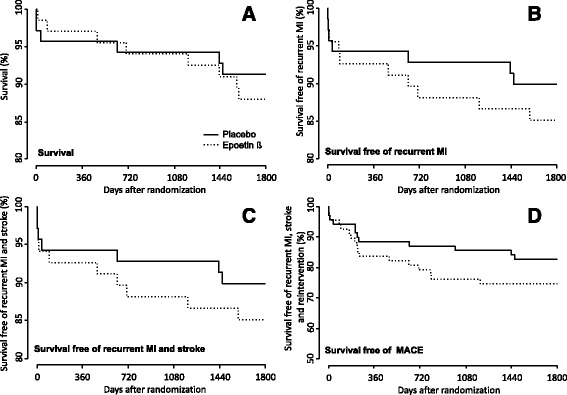
Kaplan-Meier-Curves showing the cumulative event rates according to Epoetin beta therapy or Placebo. A Analysis of survival. B Analysis of survival free of recurrent myocardial infarction (MI). C Analysis of survival free of recurrent MI and stroke. D Analysis of survival free of MACE (recurrent MI, stroke and reintervention)

Table 2 summarizes the major clinical events registered after hospital discharge in both patient groups over the extended follow-up. A total of 14 patients (10%) died during the 5-years study period, 8 (11.8%) in the epoetin-ß and 6 (8.6%) in the control group (*p* = 0.53; Fig. 1a). While 2 epoetin-ß patients and 3 placebo patients had died during the initial 6 month follow up, 6 patients receiving epoetin-ß and 3 patients receiving placebo died between 6 month and 5 years. Individual causes of death are shown in Table 3.

**Table 2:** Summary of major clinical events registered after hospital discharge in both patient groups over the 5-year follow-up

	EPO (*n* = 68)	Placebo (*n* = 70)	
Death; n(%)	8 (11.8)	6 (8.6)	*p* = 0.53
MI; n(%)	4 (5.9)	2 (2.9)	*p* = 0.38
Death or MI; n(%)	10 (14.7)	7 (10.0)	*p* = 0.40
Stroke; n(%)	1 (1.5)	0 (0)	*p* = 0.31
Death or MI or Stroke; n(%)	10 (14.7)	7 (10.0)	*p* = 0.40
Coronary bypass surgery; n(%)	1 (1.5)	0 (0)	*p* = 0.31
Target lession revascularization; n(%)	15 (22.1)	9 (12.9)	*p* = 0.15
MACE; n(%)	17 (25.0)	12 (17.1)	*p* = 0.26

**Table 3:** Summary of patients who died during the 5 year follow up period

Patient #	Group	Cause of death
1	Placebo	cardiogenic shock
2	Placebo	cardiogenic shock
3	EPO	lung embolism
4	Placebo	sudden cardiac death
5	EPO	septic shock, stroke
6	EPO	cancer
7	Placebo	after orthopedic surgery
8	EPO	sudden cardiac death
9	EPO	unknown
10	Placebo	cancer
11	EPO	sudden cardiac death
12	Placebo	unknown
13	EPO	unknown
14	EPO	unknown

Patient # 1–5 died 1–186 days after randomization. Patient # 7–14 died 187–1860 days after randomization

Six patients (4.3%) experienced MI, 2 (2.9%) in the placebo and 4 (5.9%) in the epoetin-ß group. Only 1 (1.5%) patient in the epoetin-ß group suffered a stroke (*p* = 0.31). Coronary bypass surgery was also needed in 1 (1.5%) epoetin-ß patient and none of the control patient (*p* = 0.31). Target lesion revascularization was required in 15 patients (22.1%) treated with epoetin-ß and 9 patients (12.9%) treated with placebo (*p* = 0.15).

Figure 1b and c show the cumulative event rates of survival free of recurrent MI and survival free of recurrent MI and stroke.

The current primary outcome - the cumulative incidence of MACE 5 years after randomization - occurred in 25% (*n* = 17) of the patients assigned to epoetin-ß and 17% (*n* = 12) of the patients assigned to placebo (RR 1.5; 95% CI 0.8-3.5; *p* = 0.26; Fig. 1d).

To analyze if elevated endogenous erythropoietin levels might have interfered with effects of exogenous administered epoetin-ß, we stratified the patients according to their hemoglobin level on admission. Since serum erythropoietin levels rise in an exponential manner with a decrease in hemoglobin levels [15], we analyzed clinical outcome of patients in the lower (Hb < 14,1 g/dl) and the upper (Hb > 15,5 g/dl) quartile of hemoglobin concentration on admission separately. While the lower quartile consisted of 16 control patients and 23 erythropoietin-treated patients, the upper quartile comprised 34 patients, 19 treated by placebo and 15 by erythropoietin. During the 5 years follow up 1 death in the placebo group and 5 deaths in the erythropoietin group occurred in the lower Hb-quartile (Kaplan Meier estimates of death: 6.2% placebo, 21.7% epoetin-ß; *p* = 0.19), whereas 2 control and none of the erythropoietin patients experienced death in the upper Hb-quartile (Kaplan Meier estimates of death: 10.5% placebo, 0% epoetin-ß; *p* = 0.20). The cumulative incidence of MACE 5 years after randomization occurred in 21.7% (*n* = 5) of the patients assigned to epoetin-ß and 18.8% (*n* = 3) of the patients assigned to placebo in the lower hemoglobin quartile (*p* = 0.82) and in 20% (*n* = 3) in epoetin-ß and 21.1% (*n* = 4) in placebo treated patients of the upper quartile (*p* = 0.94).

## Discussion

This extended follow-up of the REVIVAL-3 trial revealed that high-dose, short-term epoetin-ß in addition to successful PCI does not improve clinical long-term outcomes of patients with acute myocardial infarction.

To the best of our knowledge, this is currently the study with the longest follow up analyzing erythropoietin effects in STEMI patients up to 5 years. All previous trials focused on the first 6 month and to date only the large HEBE III trial has provided one year follow up results [16].

While most other trials have to deal with the problem of a selective patient inclusion with small infarct sizes the REVIVAL-3 trial only randomized large infarctions affecting approximately 27–28% of the left ventricle with impaired LV-Function [10]. This ensures, that erythropoietin effects have been tested in an adequate ischemic condition.

Prognosis of patients with STEMI remains complicated by a substantial number of death, reinfarction and heart failure. According to real life registries like the REAL register the 3-year cumulative incidence of death is about 17. 5% and MACE about 22.9% in STEMI patients treated by timely PCI with DES (drug eluting stents) [3]. Due to closely supervised and optimized therapy in the setting of RCT (randomzied controlled clinical trials) the present study has a somewhat lower however still substantial 5-year cumulative incidence of death (10%) and MACE (21%).

Since the extent of myocardial necrosis is a major determinant of adverse postinfarction-outcome, therapies able to further reduce infarct size are urgently needed. According to experimental in vivo and ex-vivo studies erythropoietin seemed to be such a promising candidate by its angiogenic, anti-inflammatory, anti-hypertrophic and anti-apoptotic properties [17]. It attenuated infarct expansion and detrimental cardiac remodeling, reduced infarct size and improved functional recovery in animal models of ischemic cardiac injury [5]. However our results are in line with the majority of clinical studies and recent meta-analyses, who all failed to demonstrate a benefit for shortterm erythropoetin therapy in PCI-treated STEMI patients in terms of both cardiac function and clinical prognosis [11, 18, 19]. A lot has been speculated about this erythropoetin paradox - why the overwhelming cardioprotective effects in animal studies could not be translated into humans.

Animal experiments were conducted in two major experimental models, MI induced by permanent ligation of a coronary artery or by temporary occlusion followed by reperfusion. In the model of permanent occlusion animals were mostly treated by a single intraperitoneal dose of 3000–5000 IU/kg of body weight erythropoietin immediately after ligation or even before [20, 21]. The best results were achieved when EPO was applied at the time of occlusion. Dose regimes in cardiac ischemia-reperfusion worked also primarily with high doses of 2500–5000 IU/kg of body weight erythropoietin intraperitoneal or intravenous and most regimes included a dose given even before ischemia was induced. Most effective results were observed when treatment was applied no later than at the time of reperfusion, i.e., 30–90 min from coronary occlusion. In contrast the majority of clinical trials did not adjust the erythropoietin dose to the individual body weight, in fact doses ranged between 30000–60000 IU, which corresponds to 430–860 IU/kg for a 70 kg patient. Drug application was carried out between 6 to 48 h in average after symptom onset [20, 21].

Therefore as a possible explanation of the erythropoietin-paradox, mostly dosing and timing of erythropoietin-administration has been supposed to be inappropriate, especially since experimental studies have shown the existence of a dose-dependent therapeutic window of time subsequent to reperfusion [22]. Beyond this window the erythropoietin induced tissue-protection is reduced or even abolished.

For example, Moon et al. showed in a rat model of permanent coronary ligation, that erythropoietin mediated cardioprotection with 3000 IU/kg of body weight was still effective when administration was delayed up to 12 h after ischemic injury, but not if the treatment was delayed for 24 h. With the lowest effective dose of 150 IU/kg of body weight beneficial effects were only observed when administered within 4 h. This efficacy was already lost when the administration was delayed by 8 h [23].

Our trial is among the studies with the highest erythropoietin doses used, nevertheless still substantial lower than those used in animal studies, and increasing the dosage further would mean increasing the risk of thromboembolic events due to elevated heamatocrit levels [24]. On the other hand, we administered erythropoietin as soon as possible in our clinical setting, namely immediately with PCI. However the average time from symptom-onset to PCI was about 250 min, exceeding the above mentioned critical time window limit of 4 h according to animal studies. Therefore, application of erythropoietin even in advance to PCI or intracoronary might be necessary to be protective and beneficial. The recently published Intra-Co-EpoMI trial however failed to demonstrate reduction of infarct size 3 months after randomized intracoronary administration of a single dose darbepoetin-alpha in STEMI patients [25]. Another novel, promising approach to increase erythropoietin doses and thereby prolong the therapeutic window without increasing the thromboembolic risk, might be the new erythropoietin derivates, which display no haematopoietic effects by preserved cardioprotection [26].

A central issue of the erythropoietin paradox however might lay in the difference between animal models and the real human world [27].

Erythropoietin mediated cardioprotective effects seem to differ across species. While cardioprotection has been clearly shown in ischemia-reperfusion models in small rodents including mouse and rabbit, experiments in larger animals such as sheep and pig were either negative or controversial [20, 21]. As mentioned above experimental studies testing erythropoietin effects in myocardial infarction mostly used healthy animals and mimicked myocardial ischemia by mechanical injury of the coronary artery. This basically contrasts the process of MI in humans. Although MI is an acute phenomenon it develops on the basis of atherosclerosis and is the final stage of this chronic complex disease. STEMI patients often experience periods of stable or unstable angina with hypoxia and/or hypoperfusion and suffer from different degrees of congestive heart failure. Therefore, they can exhibit pathologically elevated erythropoietin levels leading to erythropoietin resistance. It has been shown, that raised endogenous plasma erythropoietin concentrations in patients with congestive heart failure are associated with increased cardiovascular mortality [28]. This might also explain why we not only found no improvement of clinical outcome, but observed a trend towards an increase in MACE following epoetin beta - a trend we had already seen in the original REVIVAL 3 trial after 6 months of follow up. While 62–71% of our study patients presented with multivessel disease, in the current metaanalysis on patient level by Fokkema et al. only 36% of the patients included had multivessel disease indicating a less advanced, pronounced and preceded disease process [11]. Therefore, Epoetin-ß therapy might have encountered different endogenous erythropoietin levels, resulting in the observed adverse outcome.

Separate analysis of patients in the upper and lower quartile of baseline hemoglobin as an indirect estimate of endogenous erythropoietin levels revealed no significant impact of endogenous erythropoietin on efficiency of exogen administered epoetin-ß in terms of death and MACE - although a definitive conclusion can´t be drawn, since the event numbers are too small. However endogenous erythropoietin might not be the only confounder present. Hypertension, diabetes, aging and concomitant medication can also interfere with erythropoietin-mediated cardioprotection in clinical settings. Morphine, statins, ACE-inhibitors, angiotensin II receptor blockers, antidiabetics and clopidogrel are known to influence conditioning-induced cardioprotection and might overdrive or damp beneficial erythropoietin effects [20].

The REVEAL study by Najjar et al. on 222 patients with STEMI showed a higher incidence of death, MI, stroke and stent thrombosis upon erythropoietin use during the first 12 weeks. A subgroup analysis even revealed increased infarct size among erythropoietin patients 70 years or older [29]. Although other studies on erythropoietin in STEMI patients did not find an increased risk of adverse events over the short term, side effects of erythropoietin therapy are evident for other indications like heart failure, renal disease, anemia or cancer [30, 31]. In patients with systolic heart failure and anemia darbopoetin was accompanied by a significant increase in thrombembolic events and septic shock [32]. Side effects have been linked to erythropoietin induced increases in haematocrit, blood viscosity, blood pressure, vasoconstriction or platelet function [33]. In the present study the non-significant rise in adverse clinical events after 5 years was mainly driven by more frequent target vessel revascularization in response to epoetin beta. Corresponding quantitative coronary angiography after six months revealed an increase in segment diameter stenosis in the epoetin beta group (32 ± 19% vs. 26 ± 14%, p = 0.046). Despite a subtle induction of circulating progenitor cells by erythropoietin, the observed increase in neointima formation was not associated with progenitor cell mobilization [13]. In a rat carotid artery model of vascular injury erythropoietin induced excessive neointima formation [34]. Experimental studies in vascular lesions in mice are less clear: one study reported inhibition of neointima hyperplasia due to enhanced reendothelialisation by mobilized endothelial progenitor cells and resident endothelial cells [35], while another study described increased neointima formation upon erythropoietin treatment due to enhanced smooth muscle cell proliferation by paracrine effects of the endothelium [36]. A clinical trial, designed to analyze the effect of erythropoietin on restenosis, failed to demonstrate, that short-term ‘low-dose’ epoetin beta prevented neointimal hyperplasia in PCI-treated AMI patients [37].

Our study is limited by the fact, that the REVIVAL-3 trial was powered to detect differences in left ventricular ejection fraction and was not designed to evaluate effects on long-term clinical outcomes. Although the relatively low number of patients enrolled precludes definitive conclusions about clinical prognosis, we believe that the herein presented data can provide nevertheless valuable insights, since 97% of the study patients completed the 5-year clinical follow-up and it´s to date the only study providing clinical outcome data more than 12 month after epoetin treatment in AMI.

## Conclusion

These 5 years follow-up data show that short-term use of 3 IV doses epoetin beta in PCI-treated STEMI patients does not improve clinical long-term prognosis. Our results further support the erythropoietin paradox and advise caution regarding the application of erythropoetin in patients with STEMI.

## Abbreviations

REVIVAL: Regenerate Vital Myocardium by Vigorous Activation of Bone Marrow Stem cells
STEMI: ST-elevation myocardial infarction
MI: Myocardial infarction
PCI: Percutaneous coronary intervention
EPO: Erythropoietin
LVEF: Left ventricular ejection fraction
MACE: Major adverse cardiac events
MRI: Magnetic resonance imaging
ACVB: Coronary bypass surgery
